# *Bruguierivorax albus* gen. nov. sp. nov. Isolated from Mangrove Sediment and Proposal of *Bruguierivoracaceae* fam. nov

**DOI:** 10.1007/s00284-020-02311-w

**Published:** 2021-01-19

**Authors:** Mi Li, Kai Liu, Yonghong Liu, Chenghai Gao, Xiangxi Yi

**Affiliations:** Institute of Marine Drugs, School of Pharmaceutical Sciences, Guangxi University of Chinese Medicine, NO. 13 Wuhe Rood, Nanning, 530200 People’s Republic of China

## Abstract

A novel Gram-negative, motile, aerobic rod-shaped bacterium designated BGMRC 2031^T^ was isolated from mangrove sediment collected from Guangxi Province, China. Optimal growth occurred at 28 °C and pH 7.0–8.0 in the presence of 1% (w/v) NaCl. Alignment based on 16S rRNA gene sequences indicated that strain BGMRC 2031^T^ is most closely related to *Sodalis praecaptivus* HS1^T^ (95.6%, sequence similarity), followed by *Biostraticola tofi* DSM 19580^T^ (95.5%), *Sodalis glossinidius* DSM 16929^T^ (95.4%), and *Brenneria goodwinii* FRB141^T^ (94.9%) sequence similarity. Phylogenetic analysis based on 16S rRNA gene sequences showed that strain BGMRC 2031^T^ formed a distinct branch in a robust cluster and revealed that strain BGMRC 2031^T^, genera *Biostraticola* and *Sodalis*, formed a novel family-level clade in the order *Enterobacterales*. The novel strain showed an average nucleotide similarity of 74.7%, 74.2%, and 73.1% for *S. praecaptivus* HS1^T^, *S. glossinidius* DSM 16929^T^, and *B. tofi* DSM 19580^T^, respectively. The genomes of the BGMRC 2031^T^ shared the presence of a riboflavin synthesis gene cluster. The menaquinones of strain BGMRC 2031^T^ were MK-8 and Q-8, which were similar to those of genus *Biostraticola*. The major fatty acids (> 10%) were C_16:0_ (19.9%), summed feature 2 (iso-C_16:1_ and/or C_14:0_ 3-OH, 18.10%), summed feature 3 (C_16:1_ *ω*7*c* and/or C_16:1_ *ω*6*c*, 15.3%), C_12:0_ (13.9%), C_17:0_ cyclo (11.4%), and C_14:0_ (10.4%). The main polar lipids were phosphatidyl methylethanolamine, phosphatidyl glycerol, diphosphatidyl glycerol, phosphatidyl inositol, one unidentified phospholipid, and one unknown polar lipid. The G+C content of strain BGMRC 2031^T^ was 55.4%. Strain BGMRC 2031^T^ could extend the mean lifespan and maximum lifespan of *Caenorhabditis elegans* by 4.5% and 12.5%, respectively. Overall, the results of this study indicate that BGMRC 2031^T^ is a novel species in a new genus, for which the name *Bruguierivorax albus* gen. nov. sp. nov. is proposed, and the type of strain is designated as BGMRC 2031^T^ (= NBRC 111907^T^ = KCTC 52119^T^). In addition, a novel family, *Bruguierivoracaceae* fam. nov., is proposed to accommodate the genera *Bruguierivorax*, *Biostraticola*, and *Sodalis*.

**Supplementary information:**

The online version of this article (10.1007/s00284-020-02311-w) contains supplementary material, which is available to authorized users.

## Introduction

The order *Enterobacterales* is a large group within the class *Gammaproteobacteria* which was revised in 2016 by Adeolu et al*.* [1]. This order is characterized by its non-spore-forming, rod-shaped bacteria, as well as its Gram-negative and facultatively anaerobic characteristics [1]. At the time of its revision, the *Enterobacterales* comprised seven families according to the EzTaxon database, *Enterobacteriaceae*, *Erwiniaceae*, *Pectobacteriaceae*, *Yersiniaceae*, *Hafniaceae*, *Morganellaceae*, and *Budviciaceae* [1].

The genera *Biostraticola* and *Sodalis* were first proposed by Verbarg et al*.* [2] and Dale et al*.* [3], respectively. In 2016, *Biostraticola* and *Sodalis* were affiliated to two families, *Enterobacteriaceae* and *Pectobacteriaceae*, respectively, based on their genome phylogeny and taxonomy [1]. At the time of writing, the genus *Biostraticola* contained a single species, *B. tofi*, while *Sodalis* contained two validly named species (*S. praecaptivus* [4] and *S. glossinidius* [3]) and two candidatus species (Candidatus *S. melophagi* [5] and Candidatus *S. baculum* [6]). Two Candidatus strains were live in symbiosis with various groups of insects and, respectively, symbiose relationships with *Melophagus ovinus* and *Hemipteran* insects. To date, the majority of *Sodalis* members have been found in several insect groups, and *S. praecaptivus* has been found in human hand wounds [4, 6]. The strain *S. lignotolerans* 159R was isolated from an anaerobic lignin degrading consortium.

In our study of microbial biodiversity in medicinal mangrove plants, strain BGMRC 2031^T^ was isolated from a *Bruguiera gymnorrhiza* rhizosphere soil sample. Comparative 16S rRNA gene sequence analysis showed that strain BGMRC 2031^T^ was closely related to species in the genera *Biostraticola* (95.5%) and *Sodalis* (95.4–95.6%). However, strain BGMRC 2031^T^ could not be assigned to any species of the genera *Biostraticola* or *Sodalis* because of its low sequence similarity with the two type strains (≤ 95.6%). Therefore, the present study is conducted to report the taxonomic characterization of the new isolate, BGMRC 2031^T^.

## Materials and Methods

### Bacterial Strain and Culture Conditions

Strain BGMRC 2031^T^ was isolated from sediment of *Bruguiera gymnorrhiza* roots collected from Guangxi Province, China (21° 55′ N, 108° 50′ E). Samples were immediately stored in sterile plastic bags at 4 °C, then transported to the laboratory within 12 h. Soil (2 g) was added to 20 mL of sterilized seawater, then shaken at 37 °C for 1 h. Next, 1 mL of the suspension was transferred to 9 mL sterilized sea water and serially diluted to 10^–2^, 10^–3^, 10^–4^, and 10^–5^. The serial dilutions of the samples (200 μL) were subsequently plated onto R2A medium (0.5 g yeast extract powder, 0.5 g peptone, 0.5 g casein hydrolysate, 0.5 g glucose, 0.5 g starch, 0.3 g K_2_HPO_4_, 0.024 g MgSO_4_, 0.3 g sodium pyruvate, 15.0 g agar, 1 L seawater, pH 7.2), then incubated at 28 °C for 1 week. Single colony was selected and purified on modified Yeast Malt Extract (ISP2) (2.0 g yeast extract, 2.0 g malt extract, 2.0 g D-( +)-glucose anhydrous, 15.0 g agar powder and 1 L seawater) at 28 °C. Strain BGMRC 2031^T^ was isolated using the preceding method, then preserved in 20% (v/v) glycerol suspensions at -80 °C. The reference strain, *B. tofi* DSM 19580^T^, was obtained from the Leibniz Institut DSMZ-German Collection of Microorganisms and Cell Cultures GmbH.

### Morphological and Physiological Characteristics

Morphological and physiological characteristics were observed on modified ISP2 medium unless stated. Growth and colony morphology were observed after 2 days incubation at 28 °C. Cell morphology was observed using a scanning electron microscope (FEI Quanta 250 Environmental Scanning Electron Microscope), and the flagellum of the strain was observed via transmission electron microscopy (Hitachi Transmission Electron Microscope HT7700) after growth on ISP2 at 28 °C for 2 days. Cell motility determination was conducted by observing the development of turbidity in a tube using ISP2 semisolid medium containing 0.4% agar [7]. Gram staining was determined on ISP2 plates following the protocols described by Gerhardt et al*.* [8]. Oxidase activity was examined using 1% (w/v) *N*, *N*, *N’*, *N’*-tetramethyl-*p*-phenylenediamine reagent. Catalase activity was assessed using 3% (w/v) H_2_O_2_ solution [9]. Growth at various concentrations of NaCl (0%–15%, w/v, with an interval of 1.0%), was tested on ISP2 agar (Difco) at 28 °C. The temperature range was determined by incubating cells in ISP2 medium broth at 4 °C, 10 °C, 15 °C, 20 °C, 25 °C, 28 °C, 37 °C, 40 °C, and 45 °C for 2 weeks. The pH range for growth (pH 4.0–12.0 at intervals of 1 pH unit) was tested in ISP2 broth at 28 °C using the buffer system developed by Xu et al. [10]. Cultural characteristics were determined by observing growth of the strain at 28 °C for 2 weeks on ISP2, ISP3, ISP4, ISP5, and ISP7 agar plates, lysogeny broth (LB agar), R2A agar, and tryptic soy agar. ISCC-NBS color charts [11] were used to assess colony colors. Biochemical tests, including H_2_S production, hydrolysis of cellulose, gelatin, starch, Tweens 20, 40, and 80, were performed using the methods described by Tindall [12]. Coagulation and peptonisation of milk were evaluated as described by Gonzalez [13]. Carbohydrate metabolism was determined using API ZYM and API 20E strips (BioMérieux, Marcyl’Etoile, France) according to the manufacturer’s instructions. Anaerobic fermentation was evaluated using the API 50CH system (BioMérieux). The incubation temperature for all API kits was 28 °C, and results were observed after 48 h.

### Chemotaxonomic Characterization

Cell biomass for the chemotaxonomic characterization was obtained from ISP2 medium after incubation at 28 °C for 3 days. Polar lipids were extracted as described by Kamekura [14] and identified by two-dimensional thin-layer chromatography (TLC) on silica gel 60 GF_254_ plates (Merck KGaA, Darmstadt Germany) that had been sprayed with ethanolic molybdophosphoric acid, molybdenum blue, and ninhydrin after two-dimensional TLC [15]. Respiratory quinones were extracted and analyzed using reverse-phase HPLC [16, 17]. Cellular fatty acid composition was analyzed by gas chromatography (G6890N; Agilent Technologies, Savage, MD, USA) and identified using the Sherlock Microbial Identification System (version 6.0) according to the manufacturer’s instructions and as previously described [18].

### Phylogenetic Analyses

PCR amplification of strain BGMRC 2031^T^ with the universal primers 27F and 1492R and subsequent 16S rRNA gene sequencing [19] were conducted as described by Li et al.[20]. The purified DNA product was cloned into the pEASY-T1 vector and transformed into *Escherichia coli* DH5*α* using the pEASY-T1 cloning kit. The 16S rRNA gene sequence was compared with that of recognized species using EzBioCloud (http://www.ezbiocloud.net) [21]. Multiple alignments of the sequence data were conducted using CLUSTAL X 1.83 [22]. Phylogenetic analyses were conducted based on the neighbor-joining [23], maximum-likelihood [24], and maximum-parsimony [25] algorithms using the MEGA software (version 7.0) [26]. Kimura’s two-parameter model was used to calculate evolutionary distance matrices of the neighbor-joining method [27]. The topology of the phylogenetic tree was evaluated by bootstrap analysis with 1000 replicates [28].

### Genomic Characterization

To further distinguish strain BGMRC 2031^T^ from its closely related species, whole-genome sequencing was conducted by BGI (Wuhan, China) using the Illumina Hiseq 4000 system (Illumina, San Diego, CA, USA) according to the manufacturer’s suggested protocols. The draft genome was assembled using SOAP de novo version 2.04, and the short oligonucleotides of the obtained results were further optimized using SOAP aligner 2.21 [29, 30]. The obtained genome sequences were annotated by using the NCBI Prokaryotic Genome Annotation Pipeline and deposited at DDBJ/ENA/GenBank. Genomes were annotated using the Rapid Annotation Subsystems Technology (RAST) servers [31]. Genomic information of *B. tofi* DSM 19580^T^(SMCR00000000), *S. praecaptivus* HS^T^(CP006569.1), *S. glossinidius* DSM 16929^T^(GCA_000010085.1), and Candidatus *S. baculum* HBA(LT897836) was downloaded from GenBank and was used to evaluate genomic relatedness with strain BGMRC 2031^T^. The average nucleotide identity (ANI) was calculated using the ANI calculator tool from EzBioCloud [32]. The estimated genome sequence-based digital DNA-DNA hybridization values were calculated using formula 2 from the online Genome-to-Genome Calculator (http://ggdc.dsmz.de/ggdc.php) as described by Meier-Kolthoff et al. [33].

### Effects on Lifespan of *Caenorhabditis elegans*

The antiaging activities of crude extract of strain BGMRC 2031^T^ were investigated as previously described [34]. Briefly, the strain was fermented in ISP2 liquid medium at 28 °C and 180 rpm for 7 days. The fermentation liquor was then extracted with ethyl acetate, after which it was concentrated and desiccated to yield crude extract [35]. Wild-type *C. elegans* strains (N2) were purchased from the Caenorhabditis Genetic Center (CGC) at the University of Minnesota (Minneapolis, MN, USA). Synchronized worms can eliminate variation in results due to age differences [34]; therefore, adult worms were seeded with *E. coli* OP50 on nematode growth medium (NGM) plates and incubated for about 2 days at 20 °C. Next, M9 buffer (0.3% KH_2_PO_4_, 0.6% Na_2_HPO_4_, 0.5% NaCl, 1 mM MgSO_4_) was poured onto the plate and gently swirled it to dislodge the worms. Alkaline hypochlorite (20%) was subsequently used to completely lyse the adult worms, after which synchronized eggs were collected. The synchronized eggs were grown in M9 buffer overnight at 20 °C, then put on NGM plates at the L4 stage. Synchronized L4 larvae were subsequently used to analyze the life span of worms at 20 °C. Forty L4 stage larvae were randomly transferred onto fresh NGM plates seeded with dead *E. coli* OP50 (day 0 of lifespan), then treated with 100 μL, 0.1% (v/v) DMSO (blank control) or 500 μg·mL^−1^ BGMRC 2031^T^ crude extract. The BGMRC 2031^T^ crude extracts were dissolved in dimethyl sulfoxide, and the final concentration of DMSO was less than 0.1%. During the lifespan experiments, media were exchanged every 2 days, and survival of the animals was measured daily based on touch-provoked movement. All lifespan experiments were repeated at least two independent times.

## Results and Discussion

### Morphological and Physiological Characteristics

Colonies of strain BGMRC 2031^T^ were round, flat, and white with diameters of 0.5–1.0 mm after cultivation for 2 days on ISP2 at 28 °C. Cells of BGMRC 2031^T^ were Gram-negative and motile. Scanning electron microscopy showed that the cells were short rods of about 0.4–0.6 × 1.0–1.6 μm (Fig. S3). No growth was observed under anaerobic conditions. Strain BGMRC 2031^T^ growth occurred at 15 °C–37 °C (optimum, 28 °C) and pH 5.0–9.0 (optimum, pH 7.0–8.0) in the presence of 0%–6% (w/v) NaCl (optimum, 0–1%) (Table 1). Growth occurred on ISP2, LB, and R2A agar plates, but not ISP3, ISP4, ISP5, ISP7, or trypticase soy yeast agar plates. The strain was positive for catalase activities and negative for oxidase. Milk coagulation and peptonisation were positive, and hydrolysis of gelatin, nitrate reduction, cellulose, starch, and Tween 20, 40, and 80 were negative. The differences in the physiological and biochemical characteristics of strain BGMRC 2031^T^ and its closest related type strains are listed in Table 1 and Tables S1 and S2. Strain BGMRC 2031^T^ and the other related species were motile and catalase positive; however, strain *S. glossinidius* DSM 16929^T^ was non-motile and catalase negative. Strain BGMRC 2031^T^ was VP, valine arylamidase, cystine arylamidase, trypsin, 2-ketogluconate, and 5-ketogluconate positive, as well as positive for milk coagulation, peptonisation and fermentation of d-mannose, d-adonitol, d-glucose, dulcitol, d-sorbitol, l-fucose, d-arabinitol, and l-arabinitol. However, the strain was negative for esterase (C4) and esterase lipase (C8), as well as fermentation of d-cellobiose. These characteristics enable strain BGMRC 2031^T^ to be clearly distinguished from its closest phylogenetic relatives.

**Table 1:** Differential phenotypic characteristics of BGMRC 2031^T^ and closely related strain *Biostraticola tofi* DSM 19580^T^

Characteristic	BGMRC 2031^T^	*Biostraticola tofi* DSM 19580^T^
Colony pigmentation	White	Creamy white
Catalase/Oxidase	±	±
Temperature range for growth (°C)	15–37 (28)	5–30 (28)
pH range for growth	5.0–9.0 (7.0–8.0)	5.0–9.0 (8.0–9.0)
NaCl range for growth (%, w/v)	0–6 (0–1)	0–6 (0–1)
Milk coagulation and gelation	+	−
VP test	+	−
Sorbitol fermentation	+	−
Rhamnose	+	+
Melibiose	−	−
Esterase (C4)	−	+
Esterase lipase (C8)	−	+
Valine arylamidase	+	−
Cystine arylamidase	+	−
Trypsin	+	−
*α*-Galactosidase	−	−
*β*-Galactosidase	+	+
*N*-Acetyl-*β*-glucosaminidase	−	−
Polar lipids†	PME, PG, DPG, PI, PL, L	PME, PG, DPG, PI, PL
Respiratory quinone	MK-8 & Q-8	MK-8 & Q-8

+ , positive; − , negative, nd, not determined

^†^PME, phosphatidylmethylethanolamine; PG, phosphatidyl glycerol; DPG, diphosphatidyl glycerol; PI, phosphatidyl inositol; PL, unidentified phospholipid; L, unidentified lipid(s)

### Chemotaxonomic Characterization

The major cellular fatty acids of strain BGMRC 2031^T^ (> 10%) were C_16:0_ (19.9%), summed feature 2 (iso-C_16:1_ and/or C_14:0_ 3-OH (18.1%)), summed feature 3 (C_16:1_*ω*7*c* and/or C_16:1_*ω*6*c* (15.3%)), C_12:0_ (13.9%), C_17:0_ cyclo (11.4%), and C_14:0_ (10.4%), whereas C_17:0_ cyclo (21.0%) and C_16:0_ (20.6%) were the predominant fatty acids of strain *B. tofi* DSM 19580^T^ (Table S3). *S. glossinidius* DSM 16929^T^ was different from BGMRC 2031^T^ and *B. tofi* DSM 19580^T^ in the absence of C_19:0_ cyclo *ω*8*c*. BGMRC 2031^T^ was different from *B. tofi* DSM 19580^T^ based on the percentage of C_17:0_ cyclo and summed feature 2 (iso-C_16:1_ and/or C_14:0_ 3-OH). The C_16:0_ was main cellular fatty acid of BGMRC 2031^T^ and other neighboring families (Table 2). The major polar lipids consisted of phosphatidyl methylethanolamine, phosphatidyl glycerol, diphosphatidyl glycerol, phosphatidyl inositol, one unidentified phospholipid and one unknown polar lipid (Fig. S4). The polar lipid profile of BGMRC 2031^T^ was similar to that of *B. tofi* DSM 19580^T^, while one unknown polar lipid was detected in BGMRC 2031^T^. The menaquinones were MK-8 (60.7%) and Q-8 (39.3%), which were similar to those of *B. tofi* DSM 19580^T^ and neighboring families (Table 2).

**Table 2:** Chemotaxonomy properties of BGMRC 2031^T^ and neighboring families

Characteristic	Type genus	Catalase/Oxidase	Major cellular fatty acids	Respiratory quinone
1	*Bruguierivorax*	±	C_16:0_, feature 3 (C_16:1_ *ω*7*c* and/or C_16:1_ *ω*6*c*))	MK-8 & Q-8
2	*Pectobacterium* [38]	±	C_16:0_, summed feature 3 (C_16:1_ *ω*7*c* and/or C_16:1_ *ω*6*c*)	MK-8 & Q-8
3	*Erwinia* [39]	±	C_16:0_, summed feature 3 (C_16:1_ *ω*7*c* and/or C_16:1_ *ω*6*c*)	Q-8
4	*Escherichia* [40–42]	±	C_16:0_, summed feature 3 (C_16:1_ *ω*7*c* and/or C_16:1_ *ω*6*c*), C_17:0_ cyclo	Q-8
5	*Yersinia* [43–45]	+ /nd	C_16:0_, C_18:1_ *ω*7*c*, summed feature 3 (C_16:1_ *ω*7*c* and/or C_16:1_ *ω*6*c*)	MK-8 & Q-8
6	*Morganella* [46, 47]	nd/−	C_16:0_	nd
7	*Hafnia* [48]	±	C_16:0_ and C_17:0_ cyclo	nd
8	*Budvicia* [49]	±	C_16:0_, summed feature 3 (C_16:1_ *ω*7*c* and/or C_16:1_ *ω*6*c*)	MK-8 & Q-8

Families: 1, *Bruguierivoracaceae*; 2, *Pectobacteriaceae*; 3, *Erwiniaceae*; 4, *Enterobacteriaceae*; 5, *Yersiniaceae*; 6, *Morganellaceae*; 7, *Hafniaceae*; 8, *Budviciaceae*. + , positive; − , negative, nd, not determined

### Phylogenetic Analyses

**Fig. 1:**
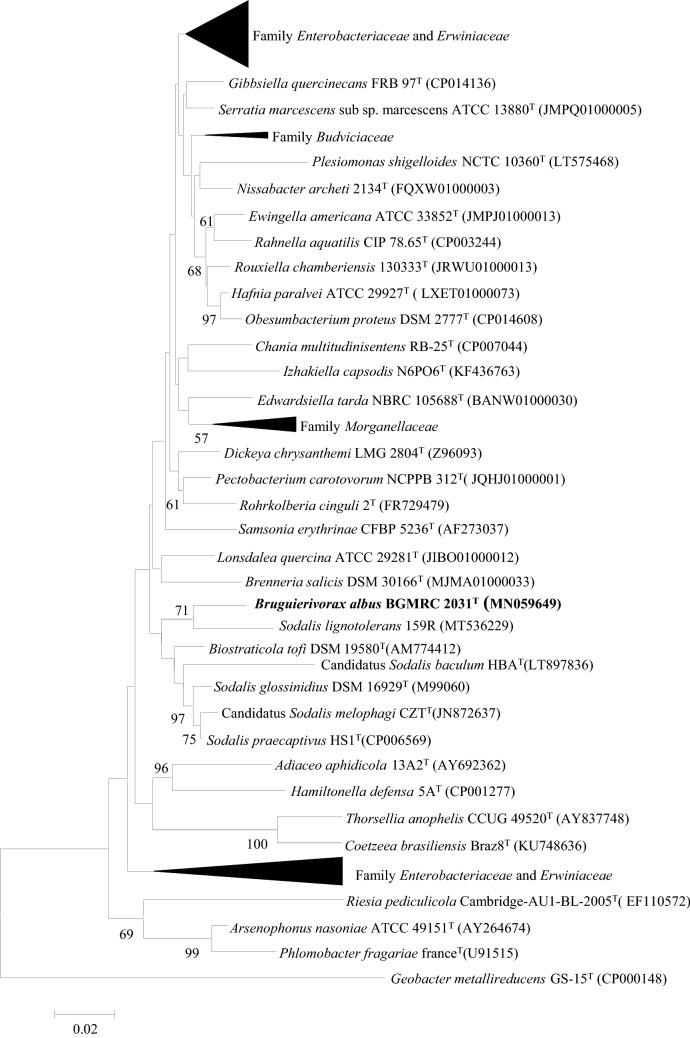
Neighbor-joining phylogenetic tree based on 16S rRNA gene sequences, showing the position of the BGMRC 2031^T^ with related taxa. The sequence of *Geobacter metallireducens* GS-15^T^ was used as an outgroup. Asterisks indicate that the corresponding branches were also recovered in trees generated with the maximum-likelihood and maximum-parsimony methods. Numbers at nodes indicate the percentage of 1000 bootstrap replicates. Only bootstrap values above 50% are shown. Bar, 0.02 substitutions per nucleotide position

The nearly complete 16S rRNA gene sequence of strain BGMRC 2031^T^ (1472 nucleotides) has been deposited in National Center for Biotechnology Information (NCBI GenBank) under accession No. MN059649. Alignment based on the 16S rRNA gene sequence in the EzBioCloud database indicated that strain BGMRC 2031^T^ is a member of the order *Enterobacterales* and showed the highest 16S rRNA gene sequence similarity to *S. praecaptivus* HS1^T^ (95.6% sequence similarity), *B. tofi* DSM 19580^T^ (95.5%), *S. glossinidius* DSM 16929^T^ (95.4%), Candidatus *S. melophagi* CZ^T^ (95.3%), Candidatus *S. baculum* HBA^T^ (91.5%), and *Brenneria goodwinii* FRB141^T^ (94.9%), suggesting that it is a novel species. This suggested that strain BGMRC 2031^T^ represented a novel species. Phylogenetic analysis based on the neighbor-joining algorithm revealed that strain BGMRC 2031^T^ and *Sodalis lignotolerans* 159R (MT536229) cluster together on a single branch. Meanwhile, Strain BGMRC 2031^T^, genera *Biostraticola* and *Sodalis* formed a distinct branch in the robust clade (Fig. 1). The maximum-parsimony and maximum-likelihood algorithms showed similar topologies and strains BGMRC 2031^T^, genera *Biostraticola* and *Sodalis* forming a separate unified cluster suggesting that BGMRC 2031^T^, *Biostraticola*, and *Sodalis* as a single novel family in the order *Enterobacterales*. (Supplementary Materials Figs. S1 and S2).

**Fig. 2:**
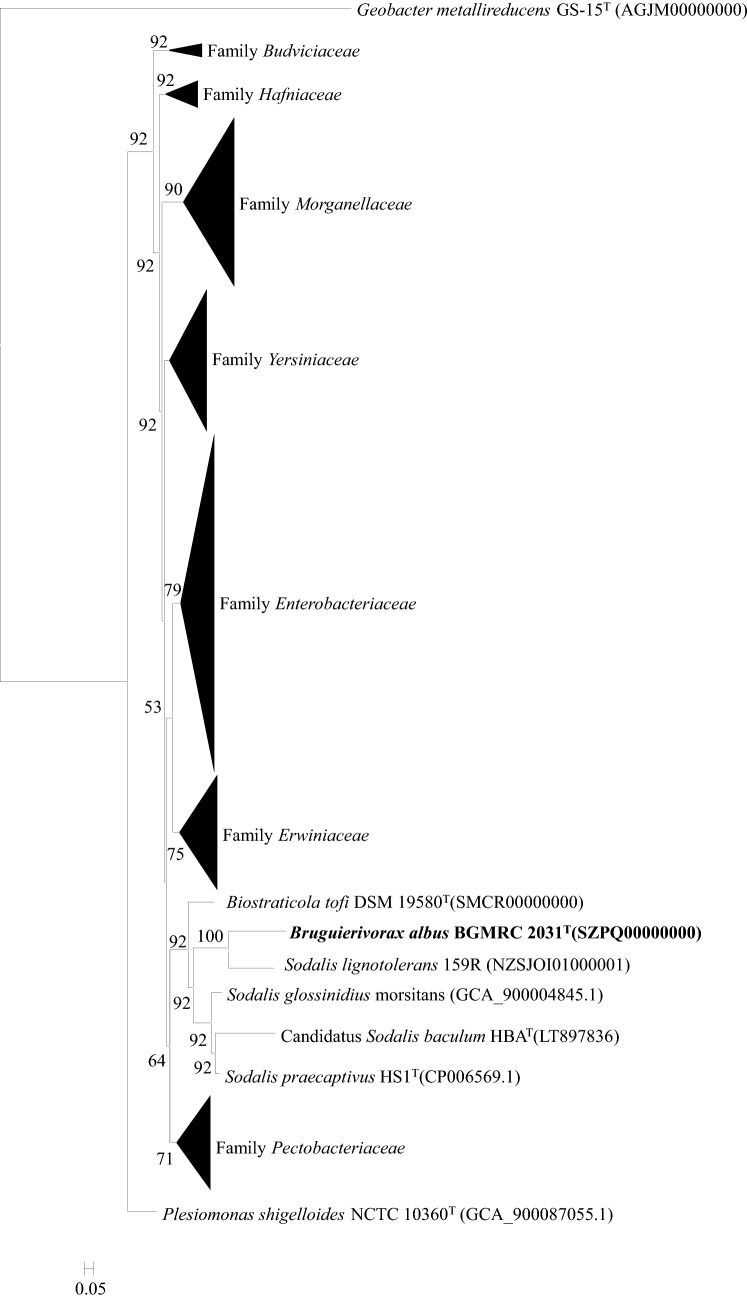
Whole-genome-based phylogenetic tree constructed using UBCGs (concatenated alignment of 92 core genes) showing the phylogenetic relationship of BGMRC 2031^T^ with reference species in the order *Enterobacterales* of *Enterobacterales*. Gene support indices (GSIs) are given at branching points. Bar, 0.05 substitution per position

The whole-genome-based phylogenetic tree was reconstructed based on the protein sequence using the up-to-date bacterial core gene set (UBCG v.3) according to its manual [36]. The tree showed that strain BGMRC 2031^T^ as well as *Biostraticola* and *Sodalis* formed an independent monophyletic clade in parallel with the species in the families *Enterobacteriaceae*, *Erwiniaceae*, *Pectobacteriaceae*, *Yersiniaceae*, *Hafniaceae*, *Morganellaceae,* and *Budviciaceae* within the order *Enterobacterales* of the class *Gammaproteobacteria* and represented a distinct family with the family *Pectobacteriaceae* and *Erwiniaceae* as the closest neighbor (Fig. 2), supporting that strain BGMRC 2031^T^ as well as the genera *Biostraticola* and *Sodalis* represented a family-level taxon.

### Genomic Characterization

The draft genome sequences of *B. tofi* DSM 19580^T^, *S. praecaptivus* HS1^T^, and *S. glossinidius* DSM 16929^T^ were obtained from NCBI (Table 3). The genome sequencing depth of strain BGMRC 2031^T^ was 199 × , and its N50 and L50 values were 147,949 bp and 12, respectively. Compared with the reference strain, the largest genome size was observed for strain BGMRC 2031^T^ (5.66 Mb). The DNA G+C content of strain BGMRC 2031^T^ was determined to be 55.4 mol%, which was higher than that of *B. tofi* DSM 19580^T^ (53.9%) and *S. glossinidius* DSM 16929^T^ (54.4%), but lower than that of *S. praecaptivus* HS1^T^ (57.1%), Candidatus *S. baculum* HBA, and *S. lignotolerans* 159R. The ANI values between strain BGMRC 2031^T^ and *B. tofi* DSM 19580^T^, *S. praecaptivus* HS1^T^, *S. glossinidius* DSM 16929^T^, Candidatus *S. baculum* HBA, and *S. lignotolerans* 159R were 73.1%, 74.7%, 74.2%, 71.24%, and 77.69%, respectively, which are below the standard ANI criteria for prokaryotic species identity (95–96%) [37]. The DDH estimated values between strain BGMRC 2031^T^ and *B. tofi* DSM 19580^T^, *S. praecaptivus* HS1^T^, *S. glossinidius* DSM 16929^T^, Candidatus *S. baculum* HBA and *S. lignotolerans* 159R were 20.5%, 21.1%, 21.1%, 26.4%, and 26.8%, respectively, which were all much lower than the standard criteria (DDH < 70%) [36]. These findings confirmed that strain BGMRC 2031^T^ represents a novel species.

**Table 3:** Genome characteristics of related strains and BGMRC 2031^T^

Characteristic	1	2	3	4	5	6
16S similarity (%)	100.0	95.49	95.56	95.42	91.28	nd
Contigs	188	32	2	4	1	1
Total length (bp)	566,116	429,087	515,942	430,208	16,224	307,533
ANI (%)	100	73.16	74.71	74.18	71.24	77.69
DDH (%)	100	29.9	26.5	26.2	26.4	26.8
N50 value (bp)	147,949	391,830	4,709	4,171,874	1,622,395	11,593
L50 values	12	4	1	1	1	80
Genome size (Mbp)	5.66	4.29	5.16	4.31	1.62	3.08
G+C content (mol%)	55.4	53.9	57.1	54.4	36.8	56.4
GenBank accession number	SZPQ00000000	SMCR00000000	CP006569.1	GCA_000010085.1	LT897836	SJOI00000000

Strains: 1, BGMRC 2031^T^; 2, *Biostraticola tofi* DSM 19580^T^; 3, *Sodalis praecaptivus* HS^T^; 4, *Sodalis glossinidius* DSM 16929^T^; 5, Candidatus *Sodalis baculum* HBA; 6, *Sodalis lignotolerans* 159R, nd, not determined

An overview of some characteristics of the respective gene content of the strain BGMRC 2031^T^, *B. tofi*, *S. praecaptivus*, *S. glossinidius*, Candidatus *S. baculum*, and *S. lignotolerans* 159R was given in Table 4. The genomes of the strain BGMRC 2031^T^ shared the presence of a riboflavin synthesis gene cluster with the strain *S. praecaptivus* HS^T^, *S. glossinidius*, and Candidatus *S. baculum*. Furthermore, the new type strains shared the lack of genes encoding soluble cytochrome b562 with those strains. Genes putatively encoded for the aminopeptidases and anaerobic respiratory reductases were only found in the genomes of the new taxon.

**Table 4:** Comparison of the presence and absence of selected genes in related strains and BGMRC 2031^T^

Genes putatively encoding	1	2	3	4	5	6
Oxidative phosphorylation/energy metabolism						
Phosphate metabolism	+	−	+	+	−	+
Anaerobic respiratory reductases	+	−	−	−	−	−
Aminopeptidases	+	−	−	−	−	+
Motility						
Flagellar motility	+	−	−	+	−	+
Electron transport chain						
Terminal cytochrome d ubiquinol oxidases	+	+	−	−	+	−
Terminal cytochrome oxidases	+	+	−	+	+	−
Biogenesis of c-type cytochromes	+	+	−	+	−	+
Other						
Trehalose biosynthesis	+	+	+	+	−	+
Denitrifying reductase gene clusters	+	−	−	+	−	+
Non-mevalonate branch of isoprenoid biosynthesis	+	−	−	−	−	+
Ammonia assimilation	+	−	−	−	-	+
Common pathway for synthesis of aromatic Compounds (DAHP synthase to chorismate)	+	−	+	+	+	−
Lysine biosynthesis DAP pathway	+	−	−	+	+	+
Riboflavin synthesis cluster	+	−	+	+	+	+
Pyridoxin (Vitamin B6) biosynthesis	+	+	+	+	+	+
Flavodoxin	+	−	+	+	+	+
Nitrogen fixation	+	−	−	+	−	+
Biotin biosynthesis	+	−	+	+	−	+
Soluble cytochrome b562	−	−	−	+	−	−

Strains: 1, BGMRC 2031^T^; 2, *Biostraticola tofi* DSM 19580^T^; 3, *Sodalis praecaptivus* HS^T^; 4, *Sodalis glossinidius* DSM 16929^T^; 5, Candidatus *Sodalis baculum* HBA; 6, *Sodalis lignotolerans* 159R

### Effects on Lifespan of *Caenorhabditis elegans*

The mean survival times (% vs DMSO) of the worms pretreated with strain BGMRC 2031^T^ and blank control are shown in Fig S5. The lifespan of worms treated with BGMRC 2031^T^ extract did not differ significantly from that of the worms treated with the blank control (0.1% DMSO), which extended the mean lifespan and maximum lifespan by 4.5% and 12.5%, respectively.

In summary, the unique phenotypic characteristics, principal fatty acid composition (C_16:0_ and iso-C_16:1_ and/or C_14:0_ 3-OH) and polar lipid composition, as well as the similar respiratory quinone composition and DNA G+C content indicated that strain BGMRC 2031^T^ may represent a novel species in a new genus of a novel family. Low 16S rRNA gene similarities (≤ 95.6%), ANI values (≤ 74.7%) and DDH values (≤ 21.1%) coupled with phenotypic and chemotaxonomic characteristics support that strain BGMRC 2031^T^ represents a novel taxon. Additionally, phylogenetic analyses indicated strain BGMRC 2031^T^ together with the genera *Biostraticola* and *Sodalis* represent a novel family within the order *Enterobacterales* of class *Gammaproteobacteria*.

### Description of *Bruguierivoracaceae* fam. nov.

*Bruguierivoracaceae* (Bru. gui. e. ri. vo. ra. ca. ce'ae. N.L. masc. n. *Bruguirerivorax* a bacterial genus; -aceae ending to denote family; N.L. fem. pl. n. *Bruguierivoracaceae* the *Bruguierivorax* family).

The major fatty acids of family *Bruguierivoracaceae* are C_16:0_ and feature 3 (C_16:1_ω7c and/or C_16:1_ ω6c). Major respiratory quinones are MK-8 and Q-8. The 16S rRNA gene-based and phylogenomic analysis showed that the genus *Bruguierivorax*, *Biostraticola* and *Sodalis* forms a separate phylogenetic clade. The family *Bruguierivoracaceae* contains the type genus *Sodalis* [3] and the genera *Biostraticola* [2] and *Bruguierivorax*. These bacteria are motile rod-shaped, catalase positive, and oxidase negative, and do not produce hydrogen disulfide. Members of this family produce acid from N-acetylglucosamine and are negative for orthinine decarboxylase lysine decarboxylase. The family *Bruguierivoracaceae* belongs to the order *Enterobacterales* of the class *Gammaproteobacteria*.

### Description of *Bruguierivorax* gen. nov.

*Bruguierivorax* (Bru.gui.e.ri.voʹrax. N.L. n. *Bruguiera* a mangrove plant genus; L. masc. adj. *vorax* devouring, ravenous, voracious; N.L. masc. n. *Bruguierivorax*, *Bruguiera* devouring).

Cells are Gram-negative, aerobic, motile, rod-shaped, catalase positive, and oxidase negative. Acid is produced from 2-ketogluconate, dulcitol, d-adonitol and d-mannose. The major respiratory quinones are MK-8 and Q-8. The major polar lipids are phosphatidyl methyl ethanolamine, phosphatidyl glycerol, diphosphatidyl glycerol, phosphatidyl inositol, one unidentified phospholipid and one unknown polar lipid. The type species is *Bruguierivorax albus.*

### Description of *Bruguierivorax* albus sp. nov.

*Bruguierivorax albus* (al’bus. L. masc. adj. *albus* white, referring to the color of the colonies).

Cells are usually 0.4–0.6 μm wide and 1.0–1.6 μm long. After 2 days of incubation on ISP2 agar at 28 °C, colonies are circular, smooth, white and round and 0.5–1.5 mm in diameter. Noval strain grew well on ISP2 agar, LB agar and R2A agar, but no growth occurred on ISP3, ISP4, ISP5, ISP7, nutrient agar or trypticase soy yeast agar plates. Optimum growth occurred at 28 °C, at pH 7.0–8.0 and in the presence of 0–1% (w/v) NaCl. The strain was negative for gelatin hydrolysis, nitrate reduction, hydrolysis of cellulose, starch, and Tween 20, 40, and 80 tests, while it was positive for milk coagulation and peptonisation tests. The strain was positive for O-nitrophenyl-*β*-D-galactopyranoside, VP, glucose fermentation, mannitol fermentation, sorbitol fermentation, amygdalin, rhamnose, and NO_2_. The alkaline phosphatase, leucine arylamidase, valine arylamidase, cystine arylamidase, trypsin, acid phosphatase, naphthol-AS-BI-phosphohydrolase and *β*-galactosidase activities were positive of new strain. New strain produced acid from d-arabinitol, l-arabinitol, d-adonitol, 5-ketogluconate, 2-ketogluconate, d-glucose, d-arabinose, l-arabinose, d-mannitol, dulcitol, d-mannose, d-ribose, d-trehalose, l-fucose, d-fructose, d-sorbitol, d-galactose, l-rhamnose, d-mannose, gluconate, d-xylose and *N*-acetyl-D-glucosamine. The major fatty acids of new strain were C_16:0_, summed feature 2 (iso-C_16:1_ and/or C_14:0_ 3-OH), summed feature 3 (C_16:1_*ω*7*c* and/or C_16:1_*ω*6*c*), C_12:0_, C_17:0_ cyclo, and C_14:0_.

This type strain BGMRC 2031^T^ was isolated from the sediment of *B. gymnorrhiza* root collected from Guangxi Province (= NBRC 111907^T^ = KCTC 52119^T^). The GenBank accession number assigned for the 16S rRNA gene sequence of strain BGMRC 2031^T^ was MN059649. The Whole-Genome Shotgun project of strain BGMRC 2031^T^ has been deposited in DDBJ/ENA/GenBank under accession number SZPQ00000000.

## Supplementary information

Below is the link to the electronic supplementary material.

Supplementary information 1 (DOCX 1858 kb)
