# Supplementary material for: Bruguierivorax albus gen. nov. sp. nov. Isolated from Mangrove Sediment and Proposal of Bruguierivoracaceae fam. nov
Source: Curr Microbiol. 2021 Jan 19;78(2):856–66. doi: 10.1007/s00284-020-02311-w (PMC7864827; doi:10.1007/s00284-020-02311-w)
Supplement: Supplementary file 1 — Supplementary information 1 (DOCX 1858 kb) [file 284_2020_2311_MOESM1_ESM.docx]

***Bruguierivorax albus* gen. nov. sp. nov., isolated from mangrove sediment and proposal of *Bruguierivoracaceae* fam. nov.**

Mi Li, Kai Liu, Yonghong Liu, Chenghai Gao*, Xiangxi Yi*

**The affiliation and full institutional address:** Institute of Marine Drugs, School of Pharmaceutical Sciences, Guangxi University of Chinese Medicine, NO. 13 Wuhe Rood, Nanning 530200, People’s Republic of China

***Authors for correspondence:** Cheng Hai Gao, E-mail: [gaoch@gxtcmu.edu.cn](mailto:gaoch@gxtcmu.edu.cn)

Telephone number: 86-0771-4733509

Xiang Xi Yi, E-mail: [yixiangxi2017@163.com](mailto:yixiangxi2017@163.com)

Telephone number: 86-0771-4733509

**Supplementary Materials**

**Supplement Table S1**. Acid produced from API 50CH of BGMRC 2031^T^ and closely related species.

Strains: 1, BGMRC 2031^T^; 2, *Biostraticola tofi* DSM 19580^T^; 3, *Sodalis praecaptivus* HS^T^; 4, *Sodalis glossinidius* DSM 16929^T^. +, positive; -, negative, nd, not determined.

| **Characteristic** | **1** | **2** | **3**§ | **4**φ |
| --- | --- | --- | --- | --- |
| D-Mannose | + | - | - | - |
| L-Arabinose | + | + | + | - |
| D-Ribose | + | + | + | - |
| D-Xylose | + | + | + | - |
| D-Adonitol | + | - | - | - |
| D-Galactose | + | + | + | - |
| D-Fructose | + | + | + | - |
| D-Mannose | + | + | + | - |
| L-Rhamnose | + | + | + | - |
| Dulcitol | + | - | - | - |
| Myo-inositol | - | - | + | - |
| D-Sorbitol | + | - | + | + |
| Methyl *α*-D-glucopyranoside | - | - | + | - |
| Amygdalin | - | - | + | nd |
| Ferric citrate of aesculin | - | - | + | nd |
| D-Cellobiose | - | + | Weak | nd |
| D-Lactose | - | Weak | + | - |
| D-Melibiose | - | - | + | - |
| D-Trehalose | + | + | + | - |
| Inulin | - | - | + | nd |
| D-Melezitose | - | - | + | nd |
| D-Raffinose | - | - | + | + |
| Starch | - | - | + | - |
| Glycogen | - | - | + | - |
| D-xylitol | - | - | + | - |
| Geutiobiose | - | - | + | nd |
| D-Turanose | - | - | + | nd |
| D-lyxose | - | - | + | nd |
| D-Tagatose | - | - | + | nd |
| D-Fucose | - | - | + | - |
| L-Fucose | + | - | + | - |
| D-Arabinitol | + | - | + | nd |
| L-Arabinitol | + | - | - | nd |
| 2-Ketogluconate | + | - | - | - |
| 5-Ketogluconate | + | - | + | nd |

§Data taken from [5]

φData taken from [4]

**Supplement Table S2**. The API ZYM and API 20E test of BGMRC 2031^T^ and related strain *Biostraticola tofi* DSM 19580^T^. +, positive; -, negative, nd, not determined.

| **Characteristic** | BGMRC 2031^T^ | *Biostraticola tofi* DSM 19580^T^ |
| --- | --- | --- |
| **Enzyme activities (API**  **ZYM):** |  |  |
| Alkaline phosphatase | + | + |
| Esterase (C4) | - | + |
| Esterase lipase (C8) | - | + |
| Lipase (C14) | - | - |
| Leucine arylamidase | + | + |
| Valine arylamidase | + | - |
| Cystine arylamidase | + | - |
| Trypsin | + | - |
| Chymotrypsin | - | - |
| Acid phosphatase | + | + |
| Naphthol-ASBI-phosphohydrolase | + | + |
| *α*-galactosidase | - | - |
| *β*-galactosidase | + | + |
| *β*-glucuronidase | - | - |
| *α*-glucanase | - | - |
| *α*-glucosidase |  |  |
| *β*-glucosidase | - | - |
| N-acetyl-*β*-glucosaminidase | - | - |
| *α*-mannosidase | - | - |
| *β*-fucosidase | - | - |
| *α*-fucosidase |  |  |
| *β*-xylosidas |  |  |
| **Utilization of (API 20NE):** |  |  |
| O-Nitrophenyl-*β*-D-Galactopyranoside | + | + |
| Arginine hydrolase | - | - |
| Lysine decarboxylase | - | - |
| Ornithine decarboxylase | - | - |
| Citrate utilization test | - | - |
| Hydrogen sulfide | - | - |
| Urease test | - | - |
| Phenylalanine deaminase | - | - |
| Indole | - | - |
| VP test | + | - |
| Gelatin liquefaction | - | - |
| Glucose fermentation | + | + |
| Mannitol fermentation | + | + |
| Inositol fermentation | - | - |
| Sorbitol fermentation | + | - |
| Rhamnose | + | + |
| Sucrose fermentation | - | - |
| Melibiose | - | - |
| Amygdalin | + | + |
| Arabinose | + | + |
| Oxidase | - | - |
| NO_2_ | + | + |
| N_2_ | - | - |

**Supplement Table S3.** Cellular fatty acid compositions of strain BGMRC 2031^T^ and *Biostraticola tofi* DSM 19580^T^

Strains: 1, BGMRC 2031^T^; 2, *Biostraticola tofi* DSM 19580^T^; Both strains were grown on ISP2 agar. ND, Not detected. The major fatty acids (greater than 10 %) are shown in bold. All data were from this study.

| **Fatty acid (%)** | BGMRC 2031^T^ | *Biostraticola tofi* DSM 19580^T^ |
| --- | --- | --- |
| **Straight-chain saturated** |  | |
| C_16:0_ | **19.9** | **20.6** |
| C_12:0_ | **13.9** | **12.5** |
| C_14:0_ | **10.4** | 9.4 |
| C_13:0_ | 0.6 | 1.1 |
| C_12:0_ 3-OH | 0.2 | 1.3 |
| **Branched saturated** |  | |
| C_17:0_ cyclo | **11.4** | **21.0** |
| **Monounsaturated** |  | |
| C_19:0_ cyclo *ω*8*c* | 2.6 | 1.1 |
| Summed feature 2ǂ | **18.1** | **11.8** |
| Summed feature 3ǂ | **15.3** | **15.8** |
| Summed feature 8ǂ | 4.5 | 2.4 |

ǂSummed feature 2 contains iso-C_16:1_ and/or C_14:0_ 3-OH; Summed feature 3 contains C_16:1_ *ω*7*c* and/or C_16:1_ *ω*6*c*; Summed feature 8 contains C_18:1_ *ω*7*c* and/or C_18:1_ *ω*6*c*

**Supplement Fig.S1.** Minimum Evolution phylogenetic tree based on the 16S rRNA gene sequences of strain BGMRC 2031^T^ and related taxa. The sequence of the *Geobacter metallireducens* GS-15^T^ were used as outgroup. Numbers at nodes indicate percentages of 1000 bootstrap resamplings only values above 50% are shown.

Family *Enterobacteriaceae* and *Erwiniaceae*

*Gibbsiella quercinecans* FRB 97^T^ (CP014136)

*erratia marcescens* sub sp. marcescens ATCC 13880^T^ (JMPQ01000005)

Family *Budviciaceae*

*Plesiomonas shigelloides* NCTC 10360^T^ (LT575468)

*Nissabacter archeti* 2134^T^ (FQXW01000003)

*Ewingella americana* ATCC 33852^T^ (JMPJ01000013)

*Rahnella aquatilis* CIP 78.65^T^ (CP003244)

*Rouxiella chamberiensis* 130333^T^ (JRWU01000013)

*Hafnia paralvei* ATCC 29927^T^ ( LXET01000073)

*Obesumbacterium proteus* DSM 2777^T^ (CP014608)

*Chania multitudinisentens* RB-25^T^ (CP007044)

*Izhakiella capsodis* N6PO6^T^ (KF436763)

*Edwardsiella tarda* NBRC 105688^T^ (BANW01000030)

Family *Morganellaceae*

*Dickeya chrysanthemi* LMG 2804^T^ (Z96093)

*Pectobacterium carotovorum* NCPPB 312^T^( JQHJ01000001)

*Rohrkolberia cinguli* 2^T^ (FR729479)

*Samsonia erythrinae* CFBP 5236^T^ (AF273037)

*Lonsdalea quercina* ATCC 29281^T^ (JIBO01000012)

*Brenneria salicis* DSM 30166^T^ (MJMA01000033)

***Bruguierivorax albus* BGMRC 2031^T^ (MN059649)**

*Biostraticola tofi* DSM 19580^T^(AM774412)

Candidatus *Sodalis baculum* HBA^T^(LT897836)

*Sodalis glossinidius* DSM 16929 ^T^(M99060)

Candidatus *Sodalis melophagi* CZT^T^(JN872637)

*Sodalis praecaptivus* HS1^T^(CP006569)

*Adiaceo aphidicola* 13A2^T^ (AY692362)

*Hamiltonella defensa* 5A^T^ (CP001277)

*Thorsellia anophelis* CCUG 49520^T^ (AY837748)

*Coetzeea brasiliensis* Braz8^T^ (KU748636)

Family *Enterobacteriaceae* and *Erwiniaceae*

*Riesia pediculicola* Cambridge-AU1-BL-2005^T^( EF110572)

*Arsenophonus nasoniae* ATCC 49151^T^ (AY264674)

*Phlomobacter fragariae* france^T^(U91515)

*Geobacter metallireducens* GS-15^T^ (CP000148)

100

99

70

74

95

96

96

61

61

73

59

0.02

*Sodalis lignotolerans* 159R (MT536229)

68

*Benitsuchiphilus tojoi* Parastrachia japonensis Kanzaki (AB548050)

Family *Enterobacteriaceae* and *Erwiniaceae*

*Chania multitudinisentens* RB-25^T^ (CP007044)

*Gibbsiella quercinecans* FRB 97^T^ (CP014136)

*erratia marcescens* sub sp. marcescens ATCC 13880^T^ (JMPQ01000005)

Family *Pectobacteriaceae*

*Rohrkolberia cinguli* 2^T^ (FR729479)

*Ewingella americana* ATCC 33852^T^ (JMPJ01000013)

*Hafnia paralvei* ATCC 29927^T^ ( LXET01000073)

*Obesumbacterium proteus* DSM 2777^T^ (CP014608)

*Rouxiella chamberiensis* 130333^T^ (JRWU01000013)

*Rahnella aquatilis* CIP 78.65^T^ (CP003244)

*Plesiomonas shigelloides* NCTC 10360^T^ (LT575468)

*Lonsdalea quercina* ATCC 29281^T^ (JIBO01000012)

*Nissabacter archeti* 2134^T^ (FQXW01000003)

*Izhakiella capsodis* N6PO6^T^ (KF436763)

*Biostraticola tofi* DSM 19580^T^(AM774412)

*Sodalis glossinidius* DSM 16929 ^T^(M99060)

***Bruguierivorax albus* BGMRC 2031^T^ (MN059649)**

Candidatus *Sodalis baculum* HBA^T^(LT897836)

Candidatus *Sodalis melophagi* CZT^T^(JN872637)

*Sodalis praecaptivus* HS1^T^(CP006569)

*Rosenbergiella nectarea* 8N4^T^ (jgi.1084674)

*Phaseolibacter flectens* ATCC 12775^T^ (AB021400)

Family *Budviciaceae*

*Edwardsiella tarda* NBRC 105688^T^ (BANW01000030)

Family *Morganellaceae*

*Adiaceo aphidicola* 13A2^T^ (AY692362)

*Hamiltonella defensa* 5A^T^ (CP001277)

Family *Enterobacteriaceae*

*Ecksteinia adelgidicola* TTL4 Gosau ^T^(HQ668160)

*Ishikawaella capsulata* N/A^T^ (AB067723)

*Buchnera aphidicola* 5AT(P001161)

*Rosenkranzia clausaccus* Elasmucha putoni ^T^(AB368828)

*Purcelliella pentastirinorum* Pentastiridius leporinus^T^(FN428803)

*Geobacter metallireducens* GS-15^T^ (CP000148)

60

72

98

71

88

50

0.020

*Sodalis lignotolerans* 159R (MT536229)

67

**Supplement Fig.S2.** Maximum likelihood phylogenetic tree based on the 16S rRNA gene sequences of strain BGMRC 2031^T^ and related taxa. The sequence of the *Geobacter metallireducens* GS-15^T^ were used as outgroup. Numbers at nodes indicate percentages of 1000 bootstrap replicates; only values above 50% are shown. Bar, 0.01 substitutions per nucleotide position.


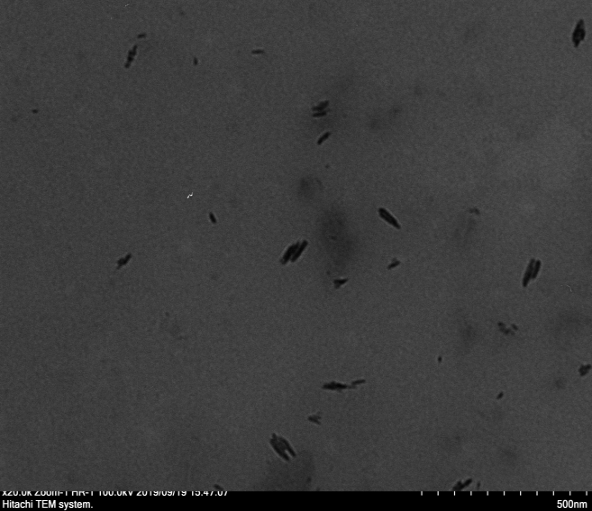

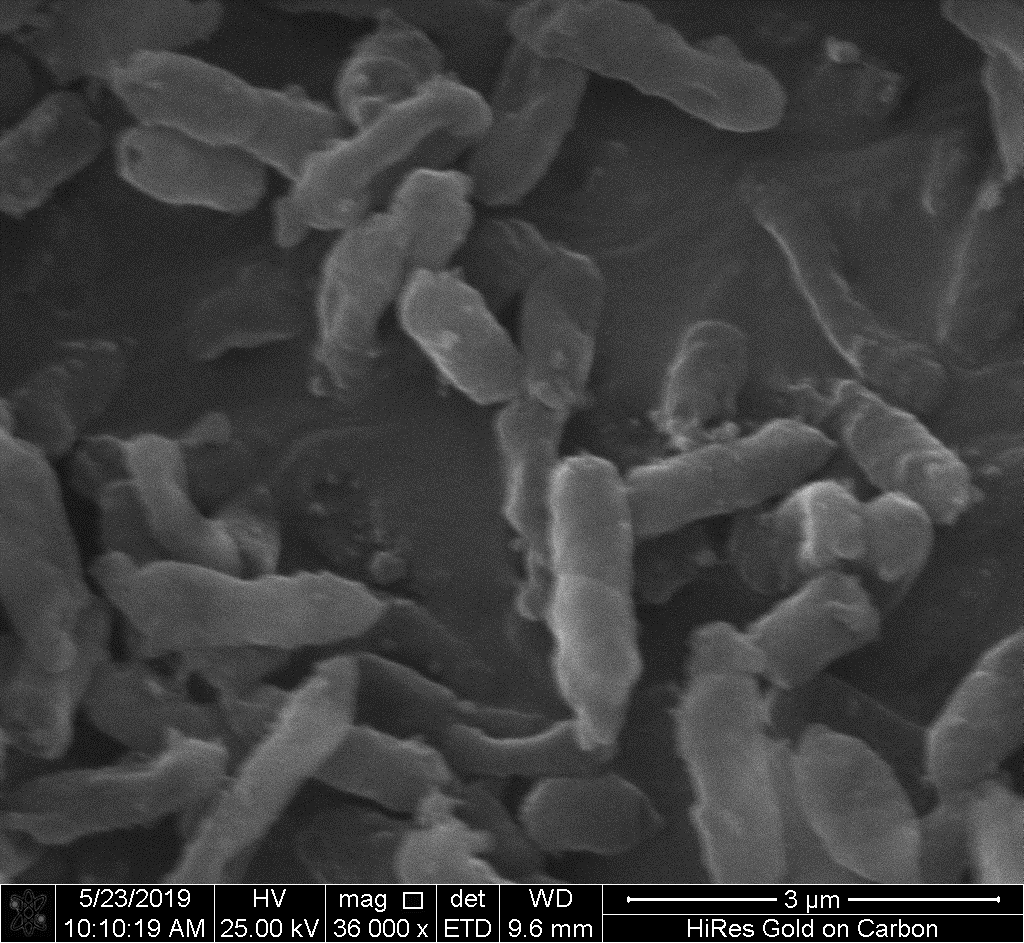


**Supplement Fig. S3.** Transmission electron micrographs and scanning electron microscopy of cells of strain BGMRC 2031^T^ grown on ISP2 agar for 2 days at 28 °C.


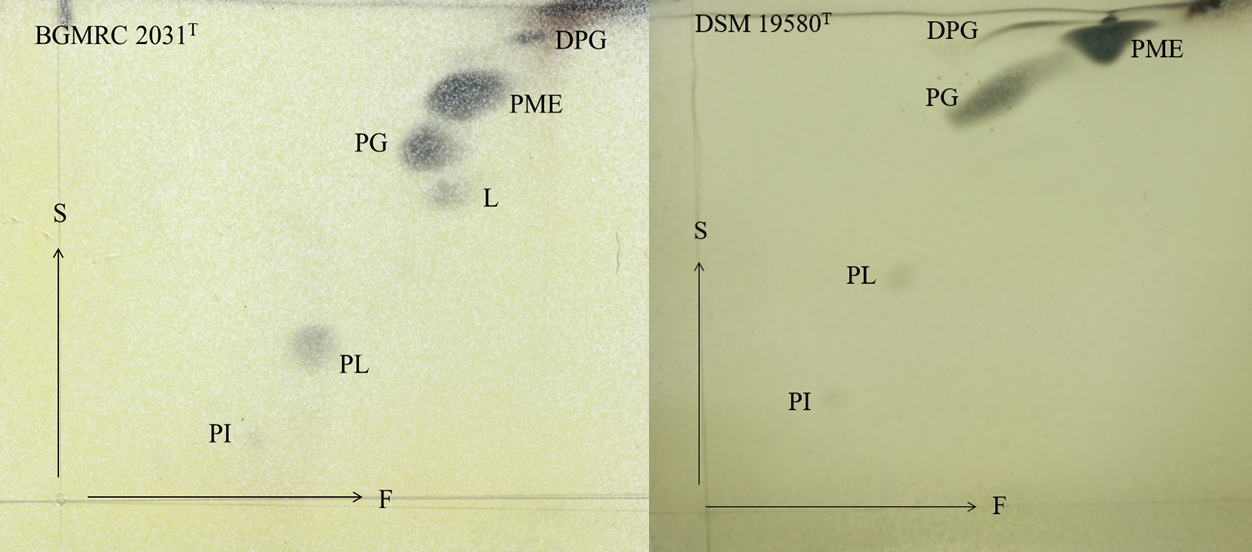


**Supplement Fig. S4.** Two-dimensional TLC patterns of the total polar lipids of strain BGMRC 2031^T^ and *Biostraticola tofi* DSM 19580^T^. diphosphatidylglycerol (DPG); phosphatidylglycerol (PG); phosphatidylmethylethanolamine (PME); unidentified phospholipid (PL); phosphatidyl inositol (PI); unidentified lipid (L). F: first dimension; S: second dimension. The solvent systems used were chloroform-methanol-water (65: 25: 4, v/v) for the first dimension and chloroform-acetic acid-methanol-water (80: 18: 12: 4, v/v) for the second dimension. Phosphomolybdic acid was used to detect all lipids.

**Supplement Fig. S5.** Effect of BGMRC 2031^T^ crude extract on lifespan of N2 worms. Mean survival time of worms treated with 500 μg•ml^-1^ of crude extract *vs.* 0.1% (v/v) DMSO control. Data are means values ± SEM, *n*=3.
